# Oral Lichenoid Reaction: An Uncommon Side Effect of Rituximab

**DOI:** 10.1155/2019/3154856

**Published:** 2019-11-06

**Authors:** Amerigo Giudice, Francesco Liborio, Fiorella Averta, Selene Barone, Leonzio Fortunato

**Affiliations:** School of Dentistry, Department of Health Sciences, Magna Graecia University of Catanzaro, Catanzaro, Italy

## Abstract

Oral lichenoid reactions (OLR) can be caused by systemic drug exposure. To the best of our knowledge, this is the second report describing a case of OLR induced by rituximab administration in a patient with a diagnosis of non-Hodgkin B-cell lymphoma. After 5 doses of rituximab, a typical pattern of OLP was identified with bilateral and symmetrical lesions on the buccal mucosa and on the right lingual margin. This temporal relationship suggested a probable association between oral lesions and drug therapy. The clinical diagnosis of a rituximab-induced OLR was confirmed by an incisional biopsy reporting a histopathological result of lichenoid mucositis consistent with oral lichen planus. Because of the increasing use of rituximab, it is necessary to know and recognize this uncommon side effect.

## 1. Introduction

Lichen planus-like lesion can be an uncommon cutaneous and mucosal adverse effect of several drugs. Despite the different etiology, it can be very challenging to distinguish oral lichenoid drug reactions (OLDRs) from oral lichen planus (OLP) with immune-mediated pathogenesis [1]. Histology can also be similar although some evidences suggest that neutrophils, eosinophils, and plasma cells can be found more in depth in drug-induced lesions [2–4].

In literature, few cases of cutaneous lichenoid reactions have been reported after administration of different anti-CD20 monoclonal antibodies [5]. Among these, rituximab is a monoclonal antibody usually used in the treatment of non-Hodgkin's lymphoma in the last 20 years [6, 7]. It is generally well-tolerated by most patients even after a long-term administration [6, 8, 9].

In this study, we present a case of rituximab-induced OLDR.

## 2. Case

A 40-year-old woman was early diagnosed with non-Hodgkin extranodal marginal-zone B-cell lymphoma of the parotid glands. She was immediately treated with 375 mg infusion of rituximab (Truxima, Mundipharma Pharmaceuticals S.r.l.) including 8 administrations: the first four doses every 15 days and the remaining ones given every month. After the 5^th^ dose the patient presented a diffuse stomatitis, together with joint pain and pink papules on her trunk and legs. A decrement of ferritin was also observed. Her past medical history was unremarkable, and before therapy, she did not report lesions or symptoms to the mouth, skin, neither genitals. Furthermore, she also did not use oral hygiene products or assume any other drug compatible with such clinical findings.

**Figure 1:**
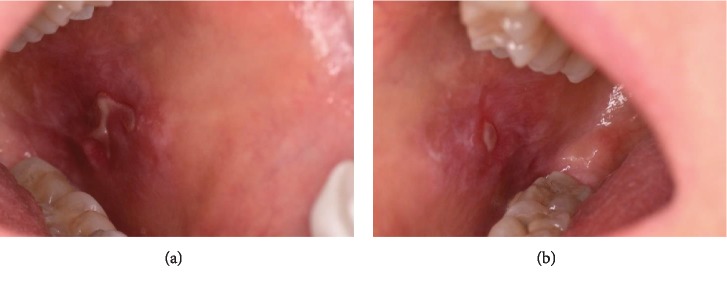
After rituximab therapy, ulcers surrounded by white reticulation and erythema on the left buccal mucosa (a), right buccal mucosa (b), can be observed.

**Figure 2:**
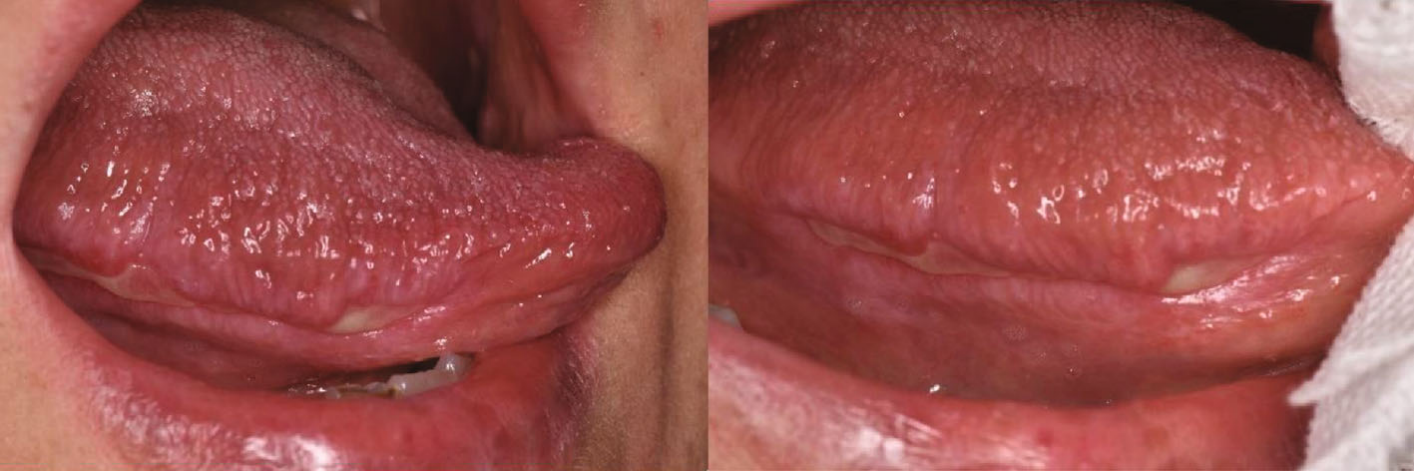
Extensive ulcer localized on the right margin of the tongue.

During the intraoral examination, carried out one month after the therapy conclusion, ulcers with white keratotic halos and peripheral erythema were found. A typical pattern of OLP was identified with bilateral and symmetrical lesions on the buccal mucosa (Figure 1) and on the right lingual margin (Figure 2).

Symptomatology was exacerbated by the assumption of certain acid foods. Based on the chronological order of the appearance of lesions, the clinical diagnosis of a rituximab-induced OLDR was confirmed by an incisional biopsy. Indeed, histopathological result reported a CD8 T-lymphocyte bandlike infiltrate close to the basal membrane, an alteration of the dermoepidermal junction and several areas of keratinocyte necrosis; few CD4 T-lymphocytes and rare CD20 B-cells were also found.

Despite the appearance of OLDR as side effects, the therapy with rituximab was effective; indeed, PET exam showed a tumor regression one month after the last administration of the drug.

The patient was treated with intralesional injections of 0.5 mL triamcinolone acetonide (Kenacort 40 mg/mL; HanAll Biopharma). The injection was carried out on both buccal mucosae once a week for 8 weeks, until the complete remission of symptomatology and almost 50% decrease in ulcer size overall. It was decided not to administer systemic corticosteroid. At 6-month follow-up, 9 months after rituximab withdrawal, oral symptomatology did not relapse; skin lesions and joint pain have autonomously healed while oral ulcers did not completely disappear.

## 3. Discussion

Oral lichenoid reactions may result from systemic drug exposure involving the skin, oral mucosa, or both. A temporal relationship between initiation of medication and onset of OLDRs may not be readily apparent: for this reason, a thorough history of systemic medication use over the preceding 12-14 months should be obtained [10–12]. OLDRs have been reported in association with many systemic medications, the most common of which include nonsteroidal anti-inflammatory drugs, antihypertensives, oral hypoglycemic agents, beta-blockers, and HIV antiretrovirals [3, 13, 14]. These lesions can be symptomatic or not, and drug withdrawal does not ensure healing.

OLDR is less common than cutaneous lichenoid drug reaction and may occur without skin involvement [3, 11]. OLDRs are common in adults but have been rarely reported in pediatric patients [15, 16].

In 2012, a case study conducted by Bakkour and Coulson reported a widespread cutaneous lichenoid eruption related to the administration of obinutuzumab, a new anti-CD20 monoclonal antibody, in a patient with relapsed follicular lymphoma previously treated with rituximab [5]. Two years later, Kuten-Shorrer et al. reported the first case of OLDR in a patient treated with rituximab for a stage IIIA follicular lymphoma [17].

Rituximab, a chimeric monoclonal antibody whose target is the B-cell CD20 antigen, is a type I monoclonal anti-CD20 antibody that has been approved for the treatment of non-Hodgkin lymphoma [18]. It showed few adverse effects, and the onset of the reaction in the reported cases ranged from 1 to 13 weeks [11, 18]. The most common side effects with rituximab intravenous administration are infusion-related alterations (such as fever, nausea, headache, chills, and rigors) while other less common reactions include infections, hypotension, hypoxia, bronchospasm, rash, and pruritus [6, 9, 11, 18].

Initially used as an antitumoral agent, rituximab became an encouraging treatment choice in B-cell-driven systemic inflammatory and autoimmune diseases (SIADs) and it is now approved for treatment of rheumatoid arthritis (RA), granulomatosis with polyangiitis (GPA), and microscopic polyangiitis (MPA) and is frequently used as off-label therapy for a wide variety of other SIADs like pemphigus vulgaris, in case of conventional treatment failure [9, 19, 20].

In this paper, the authors describe a rare case of OLDR where the rituximab dose administered was lower and shorter compared to other reports. Side effects appeared after 3 months and 5 doses totally administered. This temporal relationship suggested a probable association between oral lesions and drug administration. In that period, the patient did not receive any other antitumoral therapy. It is noteworthy that rituximab activity leads to the suppression of antigen presentation, the inhibition of T-cell recruitment, and the IFN-alpha pathway preventing, eventually, B-cell expansion [6, 12]. For this beneficial effect, recently, it has been used to treat severe and persistent lichenoid drug reaction [12, 21, 22].

Rituximab has been widely used in patients diagnosed with lymphoma, and its use is increasing in cases of a wide spread of autoimmune diseases. The authors presenting this unusual side effect believe its recognition is crucial and should not be underestimated in order to achieve a better clinical management of the patient.
